# Simulations of Infrared Reflectivity and Transmission Phonon Spectra for Undoped and Doped GeC/Si (001)

**DOI:** 10.3390/nano14171439

**Published:** 2024-09-03

**Authors:** Devki N. Talwar, Jason T. Haraldsen

**Affiliations:** 1 Department of Physics, University of North Florida, 1 UNF Drive, Jacksonville, FL 32224-7699, USA; j.t.haraldsen@unf.edu; 2 Department of Physics, Indiana University of Pennsylvania, 975 Oakland Avenue, 56 Weyandt Hall, Indiana, PA 15705-1087, USA

**Keywords:** novel binary carbides, polymorphs, zb GeC/Si (001) epilayers, surface roughness, conducting transition layer, infrared reflectivity/transmission spectroscopy

## Abstract

Exploring the phonon characteristics of novel group-IV binary XC (X = Si, Ge, Sn) carbides and their polymorphs has recently gained considerable scientific/technological interest as promising alternatives to Si for high-temperature, high-power, optoelectronic, gas-sensing, and photovoltaic applications. Historically, the effects of phonons on materials were considered to be a hindrance. However, modern research has confirmed that the coupling of phonons in solids initiates excitations, causing several impacts on their thermal, dielectric, and electronic properties. These studies have motivated many scientists to design low-dimensional heterostructures and investigate their lattice dynamical properties. Proper simulation/characterization of phonons in XC materials and ultrathin epilayers has been challenging. Achieving the high crystalline quality of heteroepitaxial multilayer films on different substrates with flat surfaces, intra-wafer, and wafer-to-wafer uniformity is not only inspiring but crucial for their use as functional components to boost the performance of different nano-optoelectronic devices. Despite many efforts in growing strained zinc-blende (zb) GeC/Si (001) epifilms, no IR measurements exist to monitor the effects of surface roughness on spectral interference fringes. Here, we emphasize the importance of infrared reflectivity $R\left(\omega \right)\;$ and transmission $T\left(\omega \right)$ spectroscopy at near normal θ*_i_* = 0 and oblique θ*_i_* ≠ 0 incidence (Berreman effect) for comprehending the phonon characteristics of both undoped and doped GeC/Si (001) epilayers. Methodical simulations of $R\left(\omega \right)$ and $T\left(\omega \right)$ revealing atypical fringe contrasts in ultrathin GeC/Si are linked to the conducting transition layer and/or surface roughness. This research provided strong perspectives that the Berreman effect can complement Raman scattering spectroscopy for allowing the identification of longitudinal optical $\omega_{\mathrm{LO}}$ phonons, transverse optical $\omega_{\mathrm{TO}}$ phonons, and LO-phonon–plasmon coupled $\omega_{\mathrm{LPP}}^{+}\;$ modes, respectively.

## 1. Introduction

Incorporating novel materials and devices into unique electronic architectures has been and still is a strong motivation for achieving the overwhelming advances and innovations in modern society. Within the technology revolution, the search for semiconductors began in the early nineteenth century when two crucial materials, silicon (Si) and germanium (Ge), were discovered [1,2]. Ever since the inception of Ge-based bipolar transistors [3,4,5,6,7,8,9,10] and the subsequent success of Si-built metal-oxide-semiconductor field effect transistors (MOSFETs) [11], many rigorous research efforts have been made to comprehend the essential characteristics of group-IV elemental (C, Si, Ge, and Sn) semiconductors. These endeavors have spearheaded spectacular technological expansion by motivating scientists and engineers to design integrated circuits (ICs), which have led to improvements in the development of complex electronic components on a single chip to create microprocessors. Currently, more than 95% of electronic devices, all over the world, are either Si-based or prepared on Si wafers. Epitaxial growth of ultrathin films on Si or Si-on-insulator (SOI) has recently offered a natural route of sustained improvement in modern state-of-the-art devices. As technological evolution continues to advance, the performance of very-large-scale integrated (VLSI) circuits and extremely matured knowledge of complementary metal-oxide semiconductors (CMOS) have revolutionized the world of electronics [12,13,14,15,16]. Due to the improved device structures with expanded functionalities and shrinkages in sizes, the need for novel wide-bandgap semiconductor materials has recently [17,18,19,20,21,22] evolved to create devices for applications in the high-temperature electronics, healthcare, photovoltaic, and automotive industries.

Despite the conceptual constraints of Si to generate light, the Si-centered optical platform has rapidly changed the landscape of photonic integrated circuits (PICs) by offering robust solutions in the areas of telecom, datacom, bio-photonics, and quantum networks [23,24,25,26,27,28,29,30,31,32,33,34,35,36,37,38,39,40,41,42,43,44,45,46], etc. In the mid-IR range, a wide variety of integrated passive and active photonic devices are instigated due to high refractive-index contrast waveguides on SOI with excellent optical properties of Si. The exploration of novel materials with ultra-low loss and high electro-optic coefficients has also been favorably examined to realize the advanced PICs with monolithically integrated light sources and efficient modulators [23,24,25,26,27,28,29,30,31,32,33,34,35,36,37,38,39,40,41,42,43,44,45,46]. The concept of achieving direct bandgap group-IV carbides is expected to offer a paradigm shift in Si-photonics concerning the monolithic implementation of light emitters. In this regard, the growth of novel group-IV binary XC (X ≡ Si, Ge, and Sn) materials and their polymorphs [e.g., 3C (cubic or zinc-blende (zb)), 2H, 4H, 6H (hexagonal), 9R, and 15R (rhombohedral) structures] on different substrates has attracted considerable attention by offering entirely new opportunities for bandgap and strain engineering [47,48,49,50,51,52,53]. Notable advances have been made in recent years due to the unique and exciting properties of group-IV materials and the broad tunability of their structural and electronic characteristics. The XC materials with high optical quality, different bandgaps $E_{g}$, hardness, high stiffness, melting point, and high thermal conductivity [47,48] are considered particularly favorable for different applications in blue/ultraviolet (UV) light-emitting diodes (LEDs), laser diodes (LDs), photodetectors, temperature sensors, wear-resistant protective coatings in IR optics, and solar cells [49,50,51,52,53], etc. Due to significant lattice mismatch and differences in the thermal expansion coefficients between the zb XC epilayers and Si substrate, one might expect the possibility of observing structural and/or intrinsic defects near the interfaces [27,28,29,30]. However, an appropriate choice of buffer layer acquiring load through relaxation of mechanical stresses in low dimensional heterostructures (LDHs) could help improve the structural qualities of epitaxially grown multi-quantum wells (MQWs) and superlattices (SLs). There remain a few intrinsic issues that might constrain the design of optoelectronic device structures. Solutions to these problems are not impossible and can be resolved by exploiting suitable experiments, e.g., growth [54,55,56,57,58,59,60,61,62,63,64,65,66,67,68,69,70,71,72] characterization [73,74,75,76,77,78,79], and evaluating their basic traits by theoretical methods [79,80,81,82,83,84,85,86,87,88,89,90,91,92,93,94,95,96,97] using state-of-the-art ab initio methodologies [98,99,100,101,102,103,104,105,106,107,108,109,110,111,112,113,114,115,116,117,118,119,120]. Clearly, these novel materials have several incredible properties that set them apart from other II-VI and III-V compound semiconductors [17,18,19,20] and make them particularly relevant for further investigations.

From an experimental standpoint, the pulsed supersonic free jets technique [54,55,56] was employed earlier for an inverse heteroepitaxial growth of Si on SiC to achieve an excellent quality of multilayer structures. A novel arc plasma C gun source has been incorporated in the molecular beam epitaxy (MBE) to grow ultrathin MQWs and SLs [57,58,59]. We have achieved a good uniformity of 3C-SiC/Si (001) epifilms using a chemical vapor deposition (CVD) method in a vertical reactor V-CVD configuration by setting different Si/C ratios and growth times from 2 min to 6 h [60]. Ultrahigh UH-CVD, reduced pressure RP-CVD, and metalorganic MO-CVD techniques are also successfully employed for preparing different Si_1−x_Ge_x_C/Si, Ge_1−x_Sn_x_C/Si, GeC/SiC, and GeC/Si epilayers [61,62,63,64,65,66,67,68,69,70,71,72]. For commercial applications, the RP-CVD method is preferred over the UH-CVD approach due to the balance between good epitaxial quality and realizing relatively high growth rates [72]. Progress made in the growth of C-based exotic materials has given opportunities to both scientists and engineers to assess valuable information about their essential traits by employing rapid and nondestructive experimental methods. The development of crystalline quality of GeC/Si (001) epifilms is a significant issue for their use in electronic devices. Raman scattering spectroscopy (RSS) and Fourier-transform infrared (FTIR) spectroscopy are widely used to characterize the major SiC (3C, 4H, 6H, 9R, 15R, etc.) polymorphs [73,74]. Between FTIR and RSS, the former technique is considered one of the fastest turnaround methods in the electronic industry for establishing phonons and the structural traits of different polar semiconductor materials (e.g., SiC/Si, GaN/Si, etc.).

In GeC/Si (001) epilayers, due to the considerable (15.5%) difference in the lattice constants between GeC film and Si substrate, one could expect the possibility of crystalline defects. Besides misfit dislocations, stacking faults, twins, and inversion domain boundaries, the most common defects in the vicinity of the GeC/Si interface are voids—also known as pits or cavities. Like SiC, such defects in GeC can be generated by Ge/C diffusion related to Ge-C nucleation. It is, therefore, necessary to find ways to identify such intrinsic impurities which could cause either an interfacial transition layer (TL) and/or surface roughness between the ‘air-epifilm’ and ‘epilyer-substrate’. Earlier, Holm et al. [106] performed IR reflectance measurements on CVD-grown 3C-SiC/Si (001) epilayers with different surface roughnesses. They suggested that rough surfaces are responsible for causing deformation in the reststrahlen band region. Other studies [107,108,109,110] also noticed high fringe contrasts below the reststrahlen band and attributed them to the existence of TL. Different theoretical models adopted in the literature [106,107,108,109,110] are inconsistent and need further scrutiny. In this context, it is reasonable to ask whether zb GeC/Si and 3C-SiC/Si (001) epilayers exhibit surface roughness and TL structures linked to native defects.

Earlier, we reported comprehensive measurements of reflectivity/transmission spectra on different V-CVD-grown 3C-SiC/Si (001) samples [60]. A careful analysis was performed exploiting a classical three-phase (air/epifilm/substrate) model by employing an effective medium approximation (EMA) to assess the bonding and structural characteristics. Comparison of the calculated results using a modified model with experimental data has provided an accurate evaluation of the film thickness d, TL d_2_, surface roughness δ, δ_2_, and electron charge carrier concentration η. In epitaxially grown GeC/Si (001) epilayers, however, no such measurements are available. On the contrary, a few reports exist where the IR absorption and μ-Raman spectroscopy studies are performed on hydrogenated amorphous GeC, *a*-Ge_1−x_C_x_:H ultrathin films prepared by radio frequency reactive magnetron sputtering methods [89,90]. Different phonon features associated with Ge-C, Ge-H, Ge-H_2_, and Ge-CH_n_ bonds have been assigned. Considering diamondoids as the building blocks of GeC SLs, both IR and RSS studies are used to investigate the impact of nanocrystal size on GeC phonon traits. In the IR measurements, a stretching Ge-C mode is ascribed to the bands appearing between ~610 cm^−1^ and 630 cm^−1^ [90]; however, for the longitudinal optical $\left(\omega_{\mathrm{LO}}\right)$ mode, a wide range of frequencies (~603 cm^−1^–812 cm^−1^) is advised for transitions from the nanostructures (diamondoids) to bulk like GeC [89]. Different theoretical calculations exist [79,80,81,82,83,84,85,86,87,88,89,90,91,92,93,94,95,96,97,98,99] using state-of-the-art ab initio methodologies [98,99]. First-principles calculation of lattice dynamics for zb GeC [83,84] provided atypical values for the transverse optical $\left(\omega_{\mathrm{TO}}\right)$ and${\;\omega }_{\mathrm{LO}}\;$ modes. A recent random structure-sampling technique with a density functional theory (DFT) offered $\omega_{\mathrm{LO}}\;$~750 cm^−1^ and $\omega_{\mathrm{TO}}\;$~630 cm^−1^ [81] phonons—consistent with our realistic rigid-ion model (RIM) calculations [118].

**Figure 1:**
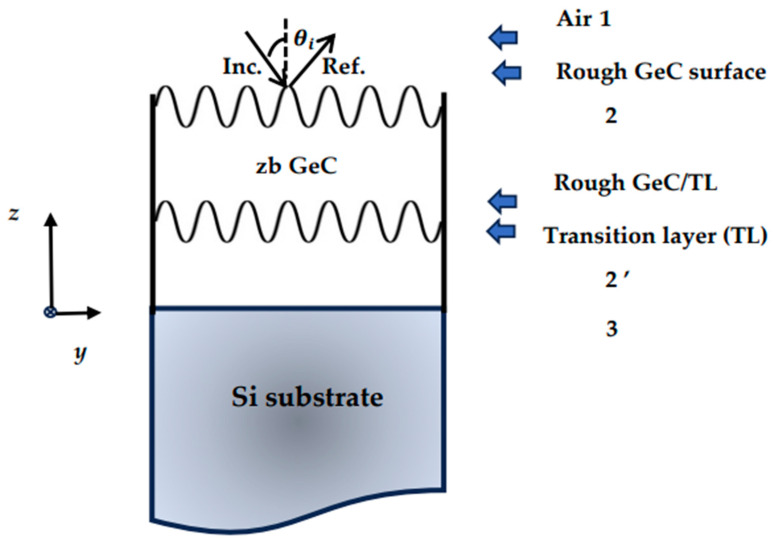
Sketch of a three-phase ideal model (‘air/epifilm/substrate’) with dielectric functions 1 air ${\tilde{\varepsilon }}_{1}=1$ (air), 2 ${\tilde{\varepsilon }}_{2}={\tilde{\varepsilon }}_{\mathrm{tf}}$ (zb GeC thin film), and 3 ${\tilde{\varepsilon }}_{3}={\tilde{\varepsilon }}_{s}$ (Si substrate) for studying the reflectance/transmission spectra of thin zb GeC/Si (001) films grown on a substrate. The modified model with the dielectric functions 1 air ${\tilde{\varepsilon }}_{1}=1$ (air), 2${\tilde{\varepsilon }}_{2}={\tilde{\varepsilon }}_{\mathrm{tf}}$ (thin film) transition layer 2′ ${\tilde{\varepsilon }}^{\prime }={\tilde{\varepsilon }}_{\mathrm{tl}}$, and 3 ${\tilde{\varepsilon }}_{3}={\tilde{\varepsilon }}_{s}$ (substrate). Scattering factors χ and χ_2_ due to roughness between GeC/air and GeC//TL surface (see Equations (8b,c)) are also included for studying the reflectivity and transmission spectra of thin films grown on a substrate [see text].

This paper aims to report methodical simulations (cf. Section 2) of the reflectivity $R\left(\omega \right)\;$ and transmission $T\left(\omega \right)\;$ spectra for GeC/Si (001) epifilms using a classical three-phase model (‘air-film-substrate’) in an effective medium approximation framework. In the ideal configuration (cf. Section 2.1.1), one expects the heterogenous structure to be perfectly smooth and optically sharp, having parallel interfaces. Due to a significant lattice mismatch of ~15.5% between GeC film and Si substrate, one expects a high density of defects, pits, and/or voids. In the modified model (cf. Figure 1), we have assumed a thin interfacial TL of thickness d_2_ near the GeC/Si (001) interface and meticulously included the roughness δ at the air–GeC surface and δ_2_ at the GeC–TL interface. Following Shokhovets et al. [116], the scattering factors χ and χ_2_ of the ripples at the interfaces (cf. Section 2.3.1) are carefully incorporated in the modified model. The predicted theoretical results of reflectivity and transmission spectra for the zb GeC/Si (001) epifilms are reported considering various settings by systematically choosing different film thicknesses d, TL d_2_, surface roughness δ and δ_2_ (cf. Section 3), and doping levels (i.e., charge carrier concentration η), etc. Earlier, by considering an intuitive physical argument, Berreman [121] has demonstrated that in the IR transmission spectrum of a freestanding thin polar film, a minimum occurs at $\omega_{\mathrm{TO}}$ frequency at the near-normal incidence (θ_i_ = 0), while in the oblique incidence (θ*_i_* ≠ 0) two minima should appear at the $\omega_{\mathrm{TO}}$ and $\omega_{\mathrm{LO}}$ phonon frequencies, respectively. Moreover, the author [121] has also argued that the $\omega_{\mathrm{TO}}$ mode arises in both the s- and p-polarizations, whereas $\omega_{\mathrm{LO}}\;$ appears only in the p-polarization. In Section 3.1, Section 3.2 and Section 3.3, we have reported our simulated results of $R\left(\omega \right)$ and $T\left(\omega \right)$ for undoped and n-doped GeC/Si (001) epifilms at near-normal θ_i_ = 0 and oblique θ_i_ ≠ 0 incidences. In heavily doped semiconductors, the presence of free charge carriers η induces strong interaction between electrons (or holes) and the optical phonons. Such interactions were studied by Raman scattering in III–V epilayers of thicknesses d > 1 μm and η > 5 E+17 cm^−3^ [73,74,75,122,123,124,125]. Experimental results were generally explained by employing two types of interactions: deformation potential and impurity-induced Fröhlich interaction. Here, we report our calculations of $\omega_{\mathrm{LO}}\;$phonon–plasmon modes near $\vec{q}\;$→ 0 in a series of heavily doped n-type GeC/Si epifilms. The results are compared/contrasted with the FTIR spectra of 3C-SiC/Si (001) epilayers. The outcomes of our theoretical descriptions are carefully examined in Section 3, with concluding remarks presented in Section 4. The methodical results of $R\left(\omega \right)$/$T\left(\omega \right)$ spectra for GeC/Si (001) epilayers offer opportunities for spectroscopists to conduct similar measurements to check our theoretical conjectures.

## 2. Theoretical Background

Like 3C-SiC/Si (001), the reflections of IR radiation in GeC/Si (001) epilayers can be described using a complex dielectric function $\tilde{\varepsilon }\left(\vec{q},\;\omega \right)$. In an ideal configuration, one expects the heteroepitaxial structures to be perfectly smooth and optically sharp with parallel interfaces. Thus, our simulation of the infrared reflectivity spectra with interference (cf. Section 2.1) caused by the reflected radiation in GeC/Si (001) epilayers is based on a multi-reflection [96] approach.

### 2.1. Model for Ideal Structure

Before considering the effects of film thickness d, charge carrier concentration η, phonon/plasmon damping, surface roughness δ, δ_2_, interfacial TL d_2_, and other factors in the GeC/Si (001) epilayer structures, we start with a conventional EMA methodology by defining the complex dielectric function $\tilde{\varepsilon }\left(\vec{q},\;\omega \right)$ for an ideal bulk material (at $\vec{q}\;$→ 0) by adopting a ‘Drude-Lorentz’ model [96]:

(1) $$\tilde{\varepsilon }\left(\omega \right)=\varepsilon_{\infty }\left[1+\frac{\omega_{\mathrm{LO}}^{2}-\omega_{\mathrm{TO}}^{2}}{\left(\omega_{\mathrm{TO}}^{2}-\omega^{2}-i\Gamma \omega \right)}\right]-\varepsilon_{\infty }\left[\frac{\omega_{p}^{2}}{\omega \left(\omega +i\gamma \right)}\right]=\left(\varepsilon_{1}+{i\varepsilon }_{2}\right)={\left(n+i\kappa \right)}^{2},$$

where ω is the frequency of incident light; $\varepsilon_{\infty }\;$ is a high-frequency dielectric constant; $\omega_{P}\left(\equiv \sqrt{\frac{4{\pi \eta e}^{2}}{{m_{e}}^{*}\varepsilon_{\infty }\;}}\right)$ is the characteristic plasma frequency of free conducting electron charge carriers which depends on its concentration η and effective mass ${m_{e}}^{*}$; γ (Γ) signifies plasma (phonon) damping coefficient; $\omega_{\mathrm{TO}}$ ($\omega_{\mathrm{LO}}$) symbolizes the TO (LO) phonon frequency near the center of the Brillouin zone (BZ) (i.e., $\vec{q}\;$→ 0); the mobility$\;\mu \left(\eta \right)$ of free charge carriers is related to $\mu \left(\eta \right)(\equiv \frac{e}{{m_{e}}^{*}\gamma \left(\eta \right)}$).

The dielectric function (cf. Equation (1)) of bulk GeC material can be separated $\tilde{\varepsilon }\left(\omega \right)\;[\equiv \varepsilon_{1}\left(\omega \right)$ + i$\varepsilon_{2}\left(\omega \right)]$ into its real [$\varepsilon_{1}\left(\omega \right)$] and imaginary [$\varepsilon_{2}\left(\omega \right)$] parts. The term$\;\varepsilon_{2}\left(\omega \right)$ represents absorption as a function of ω and affects the reflectivity (transmission) spectrum of the material. Again, $\tilde{\varepsilon }\left(\omega \right)\;$ can be related to real and imaginary parts of the complex refractive index, $\tilde{n}\left[\equiv n+i\kappa \right]=\sqrt{\tilde{\varepsilon }}$ [96]:

(2a) $$n={\left[\frac{{\left({\varepsilon_{1}}^{2}+{\varepsilon_{2}}^{2}\right)}^{1/2}+\varepsilon_{1}}{2}\right]}^{1/2}$$

(2b) $$\kappa ={\left[\frac{{\left({\varepsilon_{1}}^{2}+{\varepsilon_{2}}^{2}\right)}^{1/2}-\varepsilon_{1}}{2}\right]}^{1/2}=\left(\frac{\varepsilon_{2}}{2n}\right)$$

where n, κ in Equations (2a) and (2b) are, respectively, the index of refraction and extinction coefficients. The reflectivity $R\left(\omega \right)$ spectra of bulk zb GeC material can be expressed in terms of their reflectance coefficient $\tilde{r}\left(\omega \right)\;$ [96]:

(3) $$R\left(\omega \right)={\left|\tilde{r}\left(\omega \right)\right|}^{2}={\left[\frac{\sqrt{\tilde{\varepsilon }}-1}{\sqrt{\tilde{\varepsilon }}+1}\right]}^{2}=\left[\frac{{\left(n-1\right)}^{2}+\kappa^{2}}{{\left(n+1\right)}^{2}+\kappa^{2}}\right]\;$$

For simulating $R\left(\omega \right)\;$ of GeC/Si (001) epilayers, we have first considered an ideal system (cf. Section 2.2) in the framework of a ‘three-phase-model’, assuming an ultrathin GeC film deposited on a Si (001) substrate with an abrupt interface. Any interdiffusion that might have occurred during the growth for creating either the TL d_2_ and/or surface roughness δ, δ_2_, are systematically included in the modified model (cf. Section 2.3). Again, we have expressed here the frequencies of polar modes ($\omega_{\mathrm{TO}},$ $\omega_{\mathrm{LO}}$) and LO-plasmon coupled phonons in wave number (cm^−1^). For the GeC epilayer and Si substrate, the values of optical phonons, dielectric constants, phonon damping Γ, and effective electron mass ${m_{e}}^{*}$ are taken from the published work (see Table 1) of different research groups [77,78,80,81,86]. As the polar GeC material exhibits distinct $\omega_{\mathrm{TO}}$, $\omega_{\mathrm{LO}}$ modes and can be doped, the two terms on the right-hand side of Equation (1) are significant. On the other hand, as Si substrate is not a polar material ($i.e.,{\;\omega }_{\mathrm{TO}}\;$= $\omega_{\mathrm{LO}}=520{\;\mathrm{cm}}^{-1}$ near$\;\vec{q}$→ 0), only the free charge carriers can interact with IR radiations; hence, the first term in Equation (1) can be neglected.

#### 2.1.1. Longitudinal-Optical-Phonon–Plasmon Coupling

Raman scattering spectroscopy is commonly used to estimate the charge carrier concentration in doped semiconductors [73,74,75,76,77,122,123,124,125]. The RSS in n-doped materials depends on the electron–phonon interaction and is considered an important complement to many other spectroscopic techniques (viz., ellipsometry, luminescence, modulated spectroscopy, photoelectron spectroscopy [73,74,75,76,77,122,123,124,125], etc.). One must note that while the phonon describes an elementary excitation of lattice vibration, the plasmon represents the quantization of free electron oscillation. Again, as the lattice vibrations in LDHs are sensitive to local environments, the method can yield information about their microstructural geometry. In RSS, both polarization selection rules and intensity peak positions are sensitive to perturbations, both internal/external, such as strain, electric fields, temperature [73,74,75,76,77,122,123,124,125], etc.

In heavily doped semiconductors, including GeC, one can use a simplified approach to determine the carrier concentration η by Raman spectroscopy under non-resonant conditions. As the LO-phonon frequency approaches the plasmon frequency, their interaction instigates the so-called LO phonon–plasmon (LPP) coupled modes, providing two hybrid frequencies: $\omega_{\mathrm{LPP}}^{+}$ and $\omega_{\mathrm{LPP}}^{-}$. The $\omega_{\mathrm{LPP}}^{\pm }$ can be obtained from the singularity of the dielectric function (Equation (1), near the center of the BZ (i.e., at $\vec{q}$ → 0) by setting γ and Γ equal to zero [96]:

(4) $$\omega_{\mathrm{LPP}}^{\pm }={\left\{\frac{1}{2}\left[\omega_{\mathrm{LO}}^{2}+\omega_{P}^{2}\pm \sqrt{{\left(\omega_{\mathrm{LO}}^{2}+\omega_{P}^{2}\right)}^{2}-4\omega_{P}^{2}\omega_{\mathrm{TO}}^{2}}\right]\right\}}^{1/2}$$

where $\omega_{\mathrm{LPP}}^{+}\;$> $\omega_{\mathrm{LO}}$ and $\omega_{\mathrm{LPP}\;}^{-}$< $\omega_{\mathrm{TO}}$. From Equation (4), the carrier concentration in doped polar semiconductors can be assessed by investigating the behavior of $\omega_{\mathrm{LPP}}^{\pm }$ modes. On the other hand, the simulation of Raman intensity line shapes [125] of the $\omega_{\mathrm{LPP}}^{\pm }$ modes are much more complex. Earlier, Kukharskii [126] has theoretically studied the plasmon–phonon coupling in the most conventional GaAs. Experimentally, the observed broadening of the $\omega_{\mathrm{LO}}\;$ mode in doped GaAs increases considerably with the increase of η, while the $\omega_{\mathrm{TO}}$ phonon broadening remains nearly unaffected [125]. In doped semiconductors, the spectral line shapes of LPP modes can be calculated as follows [125]:

(5) ILPPq→,ω∝Sq→,ω Im−1ε~q→,ω 

where the response function $S\left(\vec{q},\omega \right)$ varies between different dominating scattering processes during the Raman studies, including the deformation potential with electro-optic, charge density fluctuation, impurity-induced Fröhlich mechanisms, etc. Detailed expressions involved in $S\left(\vec{q},\omega \right)\;$ for various processes can be found in [125]. In the absence of experimental Raman scattering spectroscopy data on the GeC/Si (001), our simulated $R\left(\omega \right)$ and $T\left(\omega \right)\;$ in n-doped epilayers of thickness d (μm) revealed distinct minima in the p-polarization (Berreman effect [121]). Given the epilayer thickness, the maxima (minima) are identified as the zone-center $\omega_{\mathrm{TO}}$ and the high-frequency dip as the $\omega_{\mathrm{LO}}$ phonon–plasmon coupled mode (cf. Section 3.2.5).

### 2.2. Ideal Model for GeC/Si (001) Epilayers

In zb GeC, one expects the reststrahlen band (626 cm^−1^–749 cm^−1^) of the bulk material to be well separated from the optical phonon frequency (520 cm^−1^) of the Si substrate. The simulation of IR reflectance (transmission) in the GeC/Si (001) epifilms is one of the bases of multiple reflections in epilayers for causing the interferences between the reflected (transmitted) radiations. Theoretical calculations in a ‘three-phase-model’ (see Figure 1) can be performed in both the s- and p-polarization using the dielectric functions for the air ${\tilde{\varepsilon }}_{1}=1$ (air), 2 ${\tilde{\varepsilon }}_{2}={\tilde{\varepsilon }}_{\mathrm{tf}}$ (zb GeC thin-film), and 3 ${\tilde{\varepsilon }}_{3}={\tilde{\varepsilon }}_{s}$ (Si substrate).

One must note that the s-polarized spectra combine only with the component of the dielectric function parallel to the plane of layers, while the p-polarization spectra couple simultaneously to the components parallel and perpendicular to the plane of layers. The relative contributions of the two (s- and p-) components can be determined (cf. Section 3) from the angle of incidence θ_i_. In near-normal conditions (θ_i_ ≈ 0), the total reflectance $R\left(\omega \right)$ (transmittance $T\left(\omega \right))$ can be expressed as a mean value of the s- and p-polarized reflection (transmission) coefficients [96]:

(6a) $$R\left(\omega \right)=\frac{\left|{\tilde{r}}_{123s}^{2}+{\tilde{r}}_{123p}^{2}\right|}{2}$$

(6b) $$T\left(\omega \right)=\frac{\left|{\tilde{t}}_{123s}^{2}+{\tilde{t}}_{123p}^{2}\right|}{2}$$

where ${\tilde{r}}_{123a}$ (${\tilde{t}}_{123a}$) with a (≡s- and p-) are the reflection (transmission) coefficients in the s- and p-polarization, respectively; the numbers 1, 2, and 3 signify the air, film, and substrate in a ‘three-phase model’ [96]. For an epifilm of thickness d and following Cadman and Sadowski [114], one can evaluate ${\tilde{r}}_{123a}$ (${\tilde{t}}_{123a}$) by using the following:

(7a) $${\tilde{r}}_{123a}=\frac{{\tilde{r}}_{12a}+{\tilde{r}}_{23a}\exp[i2\beta ]}{1+{\tilde{r}}_{12a}{\tilde{r}}_{23a}\exp[i2\beta ]}$$

(7b) $${\tilde{t}}_{123}=\frac{\left(1+{\tilde{r}}_{12}\right)\left(1+{\tilde{r}}_{23}\right)\exp[i2\beta ]}{1-{\tilde{r}}_{12}{\tilde{r}}_{23}\exp[i2\beta ]}$$

in terms of the Fresnel coefficients ${\tilde{r}}_{\mathrm{ija}}=\frac{{\tilde{n}}_{\mathrm{ia}}-{\tilde{n}}_{\mathrm{ja}}}{{\tilde{n}}_{\mathrm{ia}}+{\tilde{n}}_{\mathrm{ja}}}$ and phase multiplier $\beta =2\pi d\omega \sqrt{{\tilde{\varepsilon }}_{2}}$. The above approach to simulate the IR reflectivity (transmission) spectra at near-normal incidence (θ_i_ ≈ 0) in the heteroepitaxial films of thickness d can be extended to oblique incidence (θ_i_ ≠ 0) by using a methodology reported in detail elsewhere [96,112].

### 2.3. Modified Model for GeC/Si (001) Epilayers

In Equations (6a,b) and (7a,b), we have described a process for simulating the reflectivity (transmission) spectra of an ideal GeC/Si (001) structure where the ‘air/epiflm’ surface and ‘epifilm/substrate’ interface are treated as perfectly smooth and optically sharp. However, the air/epifilm surface and/or interface between film and substrate could be rough, leading to an irregular scattering of the incident radiation [96]. Thus, in a modified model, we need to meticulously include a thin conducting TL of thickness d_2_ near the GeC/Si (001) interface and incorporate (cf. Figure 1) the roughness δ near the air–GeC surface and δ_2_ between GeC–TL, respectively.

#### 2.3.1. Reflectivity and Transmission

One must note that the perception of surface roughness and ‘conducting’ TL has been suggested in many surface characterization experiments, e.g., X-ray photoelectron spectroscopy (XPS), inverse photoelectron spectroscopy (IPES), atomic force measurements (AFM), cross-sectional scanning electron microscopy (SEM), and scanning tunneling microscopy (STM) [106,119].

The Rayleigh criterion is commonly used as a test for surface roughness, giving the following critical height ($h_{c}$) of surface protuberances [116,117]:

(8a) $$h_{c}=\frac{\lambda }{{\cos\theta }_{i}}$$

where the height ($h_{c}$) of a given rough surface is defined as the minimum to maximum surface protuberance. A surface is considered smooth if h < $h_{c}$ and rough if h > $h_{c}$. The effects of roughness on IR reflectance can be described by the scattering factors χ and $\chi_{2}$. From Shokhovets et al. [116] or Landorn et al. [117], the scattering factors of possible ripples at the interfaces (cf. Figure 1) have generally followed the Gaussian distributions:

(8b) $$\chi =\exp\left[-8\;{\left(\omega \pi \delta \cos\theta_{i}\right)}^{2}\right]$$

(8c) $$\mathrm{and}\;\;\;\chi_{2}=\exp\left[-8\;{\left({\omega \pi \delta }_{2}\cos\theta_{i}\right)}^{2}\right],$$

which clearly depend upon the interfacial conditions δ, δ_2_ (rms surface roughness in μm) and wavelength λ of the incident photon. At near-normal incidence (θ_i_ ≈ 0), one can calculate the total reflection coefficients ${\tilde{r}}_{123a}$ and power reflection $R\left(\omega \right)\left(\equiv \frac{\left|{\tilde{r}}_{123s}^{2}+{\tilde{r}}_{123p}^{2}\right|}{2}\right)$ in a modified model using Equations (7a,b) and (8b,c) and following [96]:

(9) $${\tilde{r}}_{123a}=\frac{{\chi \tilde{r}}_{12a}+\chi_{2}{\tilde{r}}_{a}^{\prime }\;\exp[i2\beta ]}{1+{\chi \chi }_{2}{\tilde{r}}_{12a}{\tilde{r}}_{a}^{\prime }\;\exp[i2\beta ]}\;\mathrm{with}\;{\tilde{r}}_{a}^{\prime }=\frac{{\tilde{r}}_{22’a}+{\tilde{r}}_{2’3a}\exp[i2\beta ’]}{1+{\tilde{r}}_{22’a\;}{\tilde{r}}_{2’3a}\exp[i2\beta ’]}$$

by appropriately including the Fresnel coefficients at the GeC–TL interface along with the phase multiplier$\;\beta^{\prime \;}(\equiv 2\pi d\omega \sqrt{{\tilde{\varepsilon }}_{2\prime }}$). One must note that if the conducting TL layer thickness d_2_ and roughness δ, δ_2_ approach zero values, the modified model in the EMA transforms into an ideal case. The simulated infrared spectra of GeC/Si (001) epilayers for the ideal and modified structures are compared/contrasted (cf. Section 3) to assess the differences.

## 3. Numerical Simulations, Results, and Discussion

As stated before, an epifilm prepared on a substrate is comprised of three dielectric constants: air $\varepsilon_{\mathrm{air}}\left(=1\right)$, thin film$\;{\tilde{\varepsilon }}_{\mathrm{tf}}\left(=2\right),$ and substrate ${\tilde{\varepsilon }}_{s}\left(=3\right)$ with ‘air-film’, ‘film-substrate’, and ‘substrate-air’ interfaces. To simulate the reflectivity/transmission spectra of V-CVD-grown 3C-SiC/Si (001) samples [60], we considered earlier both the ideal (c.f. Section 2.2) and modified (c.f. Section 2.3) models [96]. The effects of surface roughness and TL played important roles in achieving very good accords with the experimental results. Without such measurements for the GeC/Si (001) epilayers, predictions are made of the $R\left(\omega \right)\;$ and $T\left(\omega \right)\;$ spectra both at near-normal (θ*_i_* = 0) and oblique incidence (θ_i_ ≠ 0) for undoped and doped films having diverse thicknesses d. In this study, we have confirmed that the Berreman effect [121] in undoped and doped thin films was complementary to Raman scattering spectroscopy for allowing the identifications of $\omega_{\mathrm{LO}}$, $\omega_{\mathrm{TO}}$ phonons as well as LO-plasmon coupled $\omega_{\mathrm{LPP}}^{+}\;$ mode, respectively. These results could possibly encourage spectroscopists to examine our theoretical conjectures.

### 3.1. Reflectivity Spectra of Semi-Infinite zb GeC

**Figure 2:**
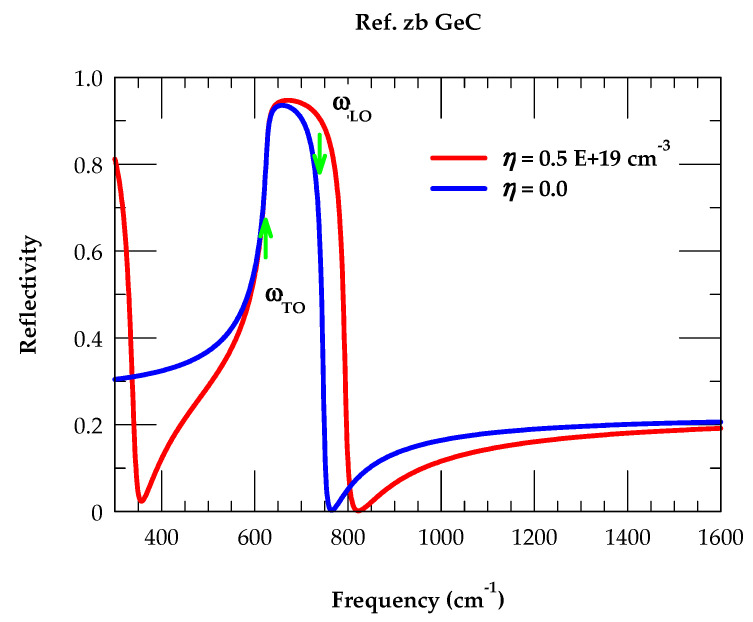
Calculated reflectance spectra at near-normal incidence for semi-infinite n-type zb GeC. The blue and red lines reflect the spectra for undoped η = 0 and n-doped with η = 0.5 E+19 cm^−3^, respectively. The positions of $\omega_{\mathrm{TO}}\;$and $\omega_{\mathrm{LO}}\;$modes of zb GeC are also marked (see text).

Using Equation (3) with appropriate values of phonons and damping parameters (cf. Table 1), we have reported in Figure 2 our simulated reflectivity spectra for undoped and doped semi-infinite GeC. The blue-colored solid line (cf. Figure 2) represents the results of an undoped GeC (η = 0), and the red-colored solid line indicates the reflectivity spectra of n-type material with the carrier concentration η = 0.5 E+19 cm^−3^. From the $R\left(\omega \right)$ spectra, we have noticed some interesting characteristics: (a) in undoped GeC, the major reststrahlen band (i.e., between $\omega_{\mathrm{TO}}$ and $\omega_{\mathrm{LO}}$) attains a maximum reflectance of ~94% near $\omega_{\mathrm{TO}}$ 626 cm^−1^, while a minimum of ~750 cm^−1^ just above $\omega_{\mathrm{LO}}\;$ maintains a ~22% value at ω > 1600 cm^−1^, and (b) for n-type GeC (with η = 0.5 E+19 cm^−3^) the spectrum indicates two changes from the carrier-free (η = 0) semi-infinite GeC material: first, the minimum above $\omega_{\mathrm{LO}}\;$(~750 cm^−1^) is shifted to a higher frequency, and secondly, another minimum appears below$\;\omega_{\mathrm{TO}}$. Obviously, these two features in $R\left(\omega \right)\;$ are linked to the charge carrier concentration and can be exploited to estimate η in doped GeC materials (cf. Section 3.2.2). Again, in the absence of reflectivity studies on bulk GeC, there exist IR absorption and μ-Raman spectroscopy reports on hydrogenated amorphous GeC, *a*-Ge_1−x_C_x_:H ultrathin films [89,90]. Using diamondoids as the building blocks of SLs, the $\omega_{\mathrm{TO}}\;$ mode ascribed to the vibration of Ge-C appearing between ~610 cm^−1^ and 630 cm^−1^ [90] is in good agreement with our reflectivity calculation.

### 3.2. Infrared Spectra of GeC/Si (001) Epifilms

In any epifilm/substrate sample, the top and bottom surfaces of the film are expected to reflect light. The total reflected light can depend on two reflections, which add up constructively or destructively depending on their phase relationship. This phenomenon is related to the wavelike nature of light, with the phase determined by the difference in optical path lengths of the two reflections. Moreover, the resulting interference pattern (fringes) at high frequency can be used to determine film thickness, provided that the refractive index and angle of incidence are known.

#### 3.2.1. Calculated Infrared Reflectance (Transmission) for Ideal GeC/Si (001)

Following the methodology outlined in Section 2.2 with parameter values from Table 1, we have reported our calculated reflectivity and transmission spectra for GeC/Si (001) epilayers. The impact of film thickness, phonon (plasma) damping, and LO phonon–plasmon coupling on the spectral profiles is carefully studied in ideal situations. In appropriate conditions, we have reported/discussed the significance of our simulated spectra of $R\left(\omega \right)\;\mathrm{and}\;T\left(\omega \right)\;$ in Section 3.2.1. (A), (A1) and (B), (B1), respectively.

(A) Reflectance Spectra: Effect of Film Thickness

**Figure 3:**
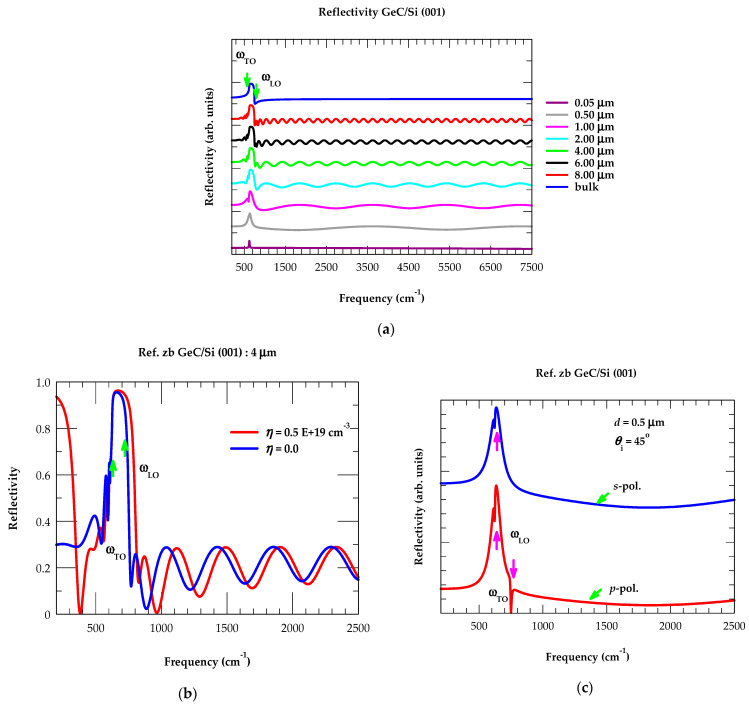
(**a**) Calculated infrared reflectance spectra at near-normal incidence θ_i_ ≈ 0 for the GeC/Si (001) epilayers of different film thicknesses. The results include bulk zb GeC as well as 8 μm, 6 μm, 4 μm, 2 μm, 1 μm, 0.5 μm, and 0.05 μm thick films. (**b**) Reflectivity spectra of 4 μm thick GeC/Si (001) epifilm, with blue- and red-colored lines indicating undoped η = 0 and n-doped η = 0.5 E+19 cm^−3^, respectively. (**c**) Polarization-dependent reflectivity of 0.5 μm thick GeC/Si (001) epifilm at oblique incident angle θ_i_ = 45°, where blue- and red-colored lines indicate s- and p-polarization spectra. The positions of $\omega_{\mathrm{TO}}\;$and $\omega_{\mathrm{LO}}\;$modes of GeC are also marked (see text).

In Figure 3a, the results of IR reflectance spectra are displayed for GeC/Si (001) epilayers with different film thicknesses, including the bulk zb GeC material. From the simulated $R\left(\omega \right)$ spectrum, we have noticed (see Figure 3a) three frequency regions of interest: (i) ω< $\omega_{\mathrm{TO}}$, (ii) between $\omega_{\mathrm{TO}}$ and $\omega_{\mathrm{LO}}$ (reststrahlen band), and (iii) $\omega >\omega_{\mathrm{LO}}$. The high reflectivity is noticed in the reststrahlen band region of the bulk material, where it reached a maximum ~94% value near $\omega_{\mathrm{TO}}\;\sim \;$626 cm^−1^, attained its minimum just above $\omega_{\mathrm{LO}}\;$~750 cm^−1^, and stayed at a nearly constant ~22% at ω > 1600 cm^−1^. In Figure 3a, the simulated $R\left(\omega \right)$ results are also displayed for zb GeC/Si (001) epilayers having different thicknesses d between 0.05 μm and 8 μm. Please note that an ultrathin film of thickness d (≡0.05 μm) reveals a sharp and narrow peak near $\omega_{\mathrm{TO}}$ with no interference fringes, because the optical path difference in the film fails to meet the required interference condition. With increasing the film thickness d between 2 μm and 8 μm, the $R\left(\omega \right)\;$spectra between the $\omega_{\mathrm{TO}}$,${\;\omega }_{\mathrm{LO}}$ region starts showing well-developed features attaining similarity to bulk-like GeC characteristics along with the interference fringes on both sides of the restsrahlen band.

In a high-frequency region, i.e., ω >> $\omega_{\mathrm{LO}}$, the contrast in the interference fringes [96] varies with the refractive indices of epifilms (n_f_) and substrates (n_s_), while the film thickness d depends upon the fringe spacing (Δω) and refractive index n_f_. In the high-frequency ω >> $\omega_{\mathrm{LO}}\;$ region, it is possible to approximately calculate the film thickness by using $d\approx {\left(2n_{f}\Delta \omega \right)}^{-1}$. For a 0.05 μm thick epifilm, our calculated value of Δω ≈ 37,000 cm^−1^ falls well beyond the simulated $R\left(\omega \right)$ spectral region with frequency, 400 cm^−1^–7500 cm^−1^, which implies that for an ultrathin film, all possible interference extrema are located outside the region of the reflectivity spectrum. As epilayer thickness increases, the corresponding extrema in $R\left(\omega \right)$ are seen shifting towards the low-frequency side with the appearance of interference contrasts. For thicker epilayers, it is more appropriate to use a generalized equation $d\approx m/2n_{f}\Delta \omega$ for obtaining the number of complete cycles m, in the frequency interval Δω. For the GeC/Si (001) epilayers, with κ_f_ = 0 and n_f_ < n_s_, the values at fringe maxima and minima can be calculated using the following [96,106]:

(10a) $$R_{\max}={\left[\frac{\left(n_{s}-1\right)}{\left(n_{s}+1\right)}\right]}^{2}$$

and

(10b) $$R_{\min}={\left[\frac{\left(n_{s}-n_{f}^{2}\right)}{\left(n_{s}+n_{f}^{2}\right)}\right]}^{2}$$

In a situation with $n_{f}$ > $n_{s}$, $R_{\max}\;$ and $\;R_{\min}$ interchange positions. Note that $R_{\max}\;$ in Equation (10a) does not depend on $n_{f}$. For ω > 1600 cm^−1^, the GeC films can be considered lossless with $n_{f}\;$= 2.7. For GeC/Si (001) epilayers and ussing the value of $n_{s}\;$ from Table 1, one can obtain $R_{\max}$= 30% and$\;R_{\min}$ = 13%, respectively.

(A1) Reflectance Spectra: Effects of Doping

In Figure 3b, we have reported the results of our calculated reflectance spectra at θ_i_ = 0 for a 4 μm thick GeC/Si (001) epifilm with η = 0 (blue-colored line: undoped) and η = 0.5 E+19 cm^−3^ (red-colored line: n-doped). Like $R\left(\omega \right)$ of semi-infinite GeC (see Figure 2), the results of n-doped GeC/Si (001) epifilm revealed two changes from the undoped spectrum (cf. Figure 3b): (a) the minimum above $\omega_{\mathrm{LO}}\;$ shifts to a higher frequency, exhibiting a slight separation in the interference fringes initially between 750 and 4000 cm^−1^, and then starts overlapping at a higher frequency of ω > 4000 cm^−1^, and (b) a minimum appears with small oscillations below $\omega_{\mathrm{TO}}$. We feel that these features can play essential roles in estimating the charge carrier concentration, η in doped epifilms.

In Figure 3c, we have reported our simulated $R\left(\omega \right)$ spectra for a 0.5 μm thick GeC/Si (001) epifilm at oblique incidence θ_i_ = 45° in the *s*- (blue-colored line) and p-polarization (red-colored line). Comparison with the results at near-normal incidence (i.e., at θ_i_ = 0, see Figure 3a) spectra has revealed the $\omega_{\mathrm{TO}}\;$ frequency in both s- and p-polarization, while $\omega_{\mathrm{TO}}\;$and$\;\omega_{\mathrm{LO}}$ modes appear only in the p-polarized spectrum (Berreman effect [121]). We feel that using the reflectivity (cf. Section 3.2.1 (A), (A1)) and/or transmission studies (cf. Section 3.2.1 (B), (B1)) at oblique incidence can allow the direct observation of $\omega_{\mathrm{LO}}$ mode in undoped epilayers and the LO-plasmon coupled $\omega_{\mathrm{LPP}}^{+}\;$ mode in doped samples (c.f. Section 3.2.5).

(B) Transmission Spectra: Effect of Film Thickness

**Figure 4:**
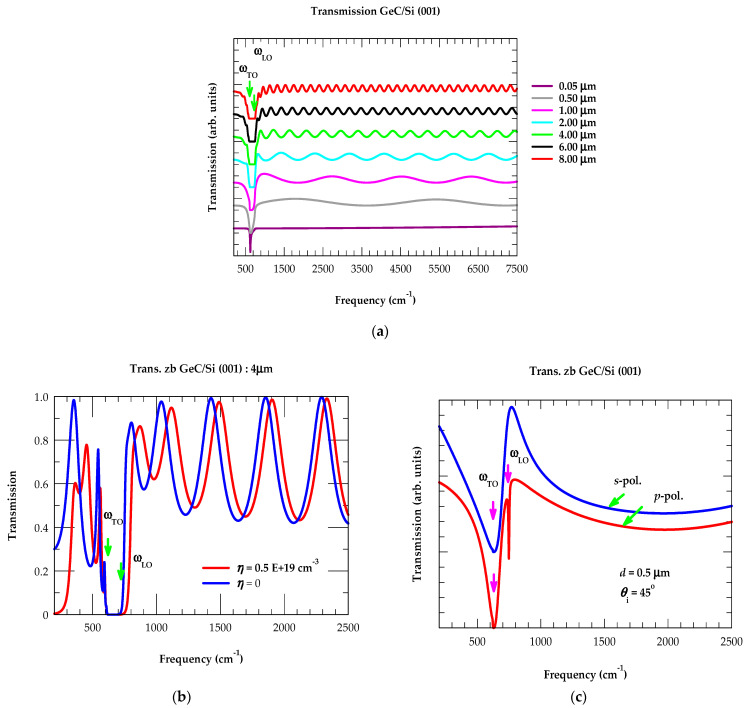
(**a**) Calculated infrared transmission spectra at near-normal incidence θ_i_ ≈ 0 for the GeC/Si (001) epilayers of different film thicknesses. The results include 8 μm, 6 μm, 4 μm, 2 μm, 1 μm, 0.5 μm, and 0.05 μm thick films. (**b**) Transmission spectra of 4 μm thick GeC/Si (001) epifilm, with blue- and red-colored lines indicating undoped η = 0 and n-doped η = 0.5 E+19 cm^−3^, respectively. (**c**) Polarization-dependent transmission spectra of 0.5 μm thick GeC/Si (001) epifilm at oblique incident angle θ_i_ = 45°, where blue- and red-colored lines indicate s- and p-polarization spectra. The positions of $\omega_{\mathrm{TO}}\;$and $\omega_{\mathrm{LO}}\;$modes of GeC are also marked (see text).

In Figure 4a, we have reported our calculated results of transmission spectra for the GeC/Si (001) epilayers with different thicknesses d (≡0.05 μm–8 μm). For ultrathin film (d ≡ 0.05 μm), the simulated $T\left(\omega \right)\;$spectra exhibited a sharp dip near $\omega_{\mathrm{TO}}$ mode frequency with no interference fringes. As the film thickness d steadily increased to 8.0 μm, the calculations revealed deep/flat bands leveling to achieve the GeC bulk-like phonon modes with two extreme edges falling between the reststrahlen band region (i.e., near $\omega_{\mathrm{TO}}\;\sim 626{\;\mathrm{cm}}^{-1}\;$ and $\omega_{\mathrm{LO}}\;\sim 750{\;\mathrm{cm}}^{-1}$) and interference fringes.

(B1) Transmission Spectra: Effects of Doping

In Figure 4b, the simulated $T\left(\omega \right)$ spectra of a 4 μm thick GeC/Si (001) epifilm are displayed at θ_i_ = 0, for η = 0 (with blue-colored line indicating undoped) and η = 0.5 E+19 cm^−3^ (with red-colored line indicating n-doped). Like $R\left(\omega \right)$, the transmission spectrum of doped epifilm revealed two modifications from the undoped results (cf. Figure 4b): (a) a minimum above $\omega_{\mathrm{LO}}$ shifts to a higher frequency with slight separation in interference fringes initially (ω<4000 cm^−1^), and the fringes start overlapping at ω > 4000 cm^−1^, and (b) for $\omega <\omega_{\mathrm{TO}}$, a minimum appears with small oscillations. In Figure 4c, we have displayed our calculated $T\left(\omega \right)$ spectra for a 0.5 μm thick GeC/Si (001) epifilm at θ_i_ = 45 ° in the s- (blue line) and p-polarization (red line). Comparison of the $T\left(\omega \right)\;$at near-normal incidence (i.e., at θ_i_ = 0, see Figure 4a) reveals $\omega_{\mathrm{TO}}\;$ mode appearing in both s- and p-polarization, while $\omega_{\mathrm{TO}}\;$ and $\;\omega_{\mathrm{LO}}$ phonons are seen in the p-polarization of the $T\left(\omega \right)$ spectrum (Berreman effect [121]). As stated before, the reflectivity and transmission studies (cf. Section 3.2.1 (A), (A1) and (B), (B1)) at oblique incidence allow direct observation of$\;\omega_{\mathrm{LO}}$ phonon in undoped epifilms and may permit perceiving the LO-plasmon coupled $\omega_{\mathrm{LPP}}^{\pm }\;$modes (cf. Section 3.2.5) in doped epilayers.

#### 3.2.2. LO-Phonon–Plasma Coupled $\omega_{LPP}^{\pm }$ Modes

**Figure 5:**
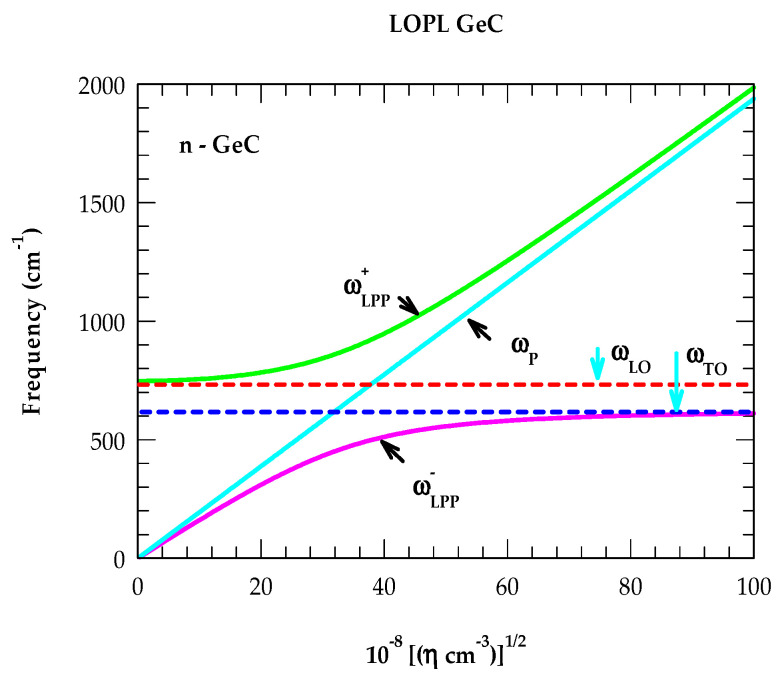
Calculated LO-phonon–plasmon coupled $\omega_{\mathrm{LPP}\;}^{\pm }$mode frequencies in n-type GeC as a function of free carrier concentration η. The values of ω_LO_, ω_TO_ modes (dotted lines) of GeC are indicated by sky blue arrows. Variation in ω_P_ (sky blue line) with η is also displayed (see text).

For n-type GeC, we have presented in Figure 5 our calculated results of the $\omega_{\mathrm{LPP}}^{\pm }$ (green color $\omega_{\mathrm{LPP}}^{+}$, magenta color $\omega_{\mathrm{LPP}}^{-}$) and ω_P_ modes (sky blue color) as a function of η. These outcomes are acquired from Equation (1) by setting the real part of the dielectric function, $\tilde{\varepsilon }\left(\omega \right)$, to zero. One must note that for a smaller value of the charge carrier concentration η, the $\omega_{\mathrm{LPP}}^{-}$ mode near $\vec{q}\;$→ 0 exhibits a plasmon-like behavior and becomes phonon-like for larger η. On the other hand, the $\omega_{\mathrm{LPP}}^{+}\;$mode with a smaller value of η lies close to $\omega_{\mathrm{LO}}$ displaying a phonon-like characteristic, which turns into a plasmon-like one at a higher charge carrier concentration η. In many doped polar semiconductors, extensive Raman scattering and FTIR spectroscopy measurements are reported [73,74,75,76,77,122,123,124,125] for identifying LO-plasma coupled phonons.

In Raman scattering spectroscopy, the relative shifts of $\omega_{\mathrm{LPP}\;}^{\pm }$ modes are observed with the broadening of their line widths by increasing η and γ. Thus, only the $\omega_{\mathrm{LPP}}^{-}$ phonons are detected with a small γ and low η. The $\omega_{\mathrm{LPP}}^{+}$ mode frequencies are perceived with significantly larger values of γ and η. Moreover, the shifts of $\omega_{\mathrm{LPP}}^{+}$ phonons to higher energy regions by increasing η cause broader widths with weaker intensities. Obviously, these observations have made the RSS measurements less sensitive [73,74,75,76,77,122,123,124,125] for extracting η at higher doping levels. In this context, we strongly feel that the study of p-polarized $R\left(\omega \right)$ [$T\left(\omega \right)$] spectra at oblique incidence (Berreman effect) [121] in doped GeC/Si (001) epifilms (cf. Section 3.2.5) will be valuable for perceiving the modes linked to $\omega_{\mathrm{LPP}}^{+}.$ In $R\left(\omega \right)$ [$T\left(\omega \right)],\;$one would also expect the $\omega_{\mathrm{LPP}}^{+}$ phonons to steadily shift towards higher-frequency regions by increasing η and to help with assessing the accurate values of charge carrier concentration.

#### 3.2.3. Impact of γ, μ, and η on ω_P_

**Figure 6:**
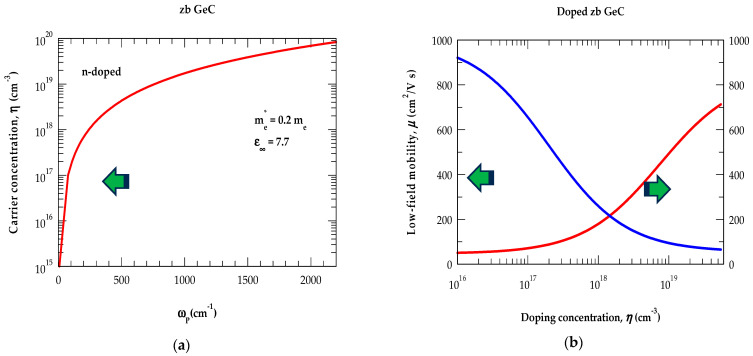
(**a**) Calculated plasma frequency *ω*_P_ in cm^−1^ versus charge carrier concentration *η* (cm^−3^) in n-type GeC. (**b**) Calculated low field mobility μ in (cm^2^/Vs) (left) and plasmon coupling coefficient γ in cm^−1^ versus charge carrier concentration *η* (cm^−3^) in n-type GeC (see text).

In doped polar materials, the plasma damping coefficient γ$\left(\eta \right)\left(\equiv \frac{e}{m^{*}\mu \left(\eta \right)}\right)\;$related to the mobility depends upon η via $\omega_{P}$. In Figure 6a, we have reported the impact of η on ω_P_ while the dependence of η on μ and γ is displayed in Figure 6b using the empirical relationship of Caughey and Thomas [127]. Consistent with the electrical measurements, our results [see Figure 6b] have revealed a reduction in μ by increasing η. Appropriate parameter values (cf. Table 2) extracted from Figure 6a,b are integrated in Equations (6a,b) and (7a,b) to monitor the effects of η, γ, and $\omega_{P}\;$on the $R\left(\omega \right)$ and $T\left(\omega \right)$ spectra for the GeC/Si (001) epilayers.

#### 3.2.4. Effects of γ, η, and ω_P_ on $R\left(\omega \right)$ and $T\left(\omega \right)$

**Figure 7:**
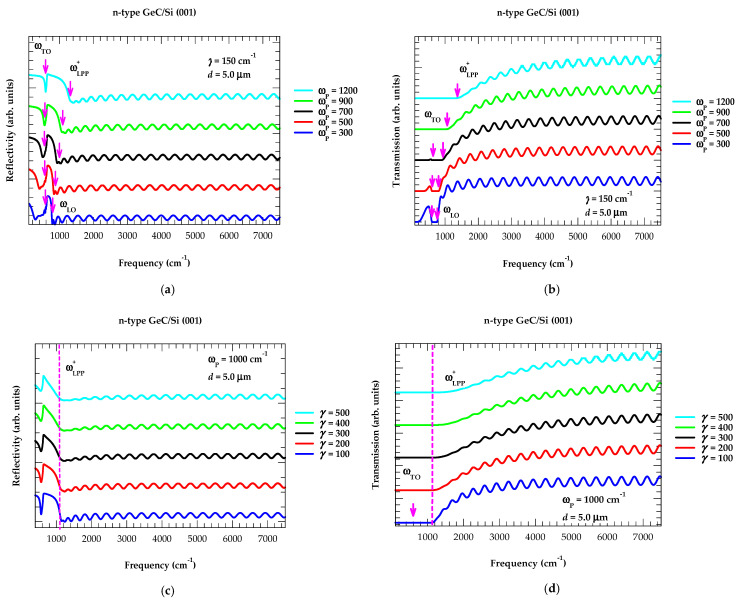
Calculated infrared spectrum of 5.0 μm thick n-type GeC/Si(100) epifilm: (**a**) Reflectivity spectra as a function of frequency (cm^−1^) for a fixed value of γ = 150 cm^−1^ while changing ω_P_ from 300, 500, 700, 900, and 1200 cm^−1^. (**b**) Same key as for (**a**) but for the transmission spectra of 5.0 μm thick n-type GeC/Si(100) epifilm. (**c**) Reflectivity spectra as a function of frequency (cm^−1^) for a fixed value of ω_P_ = 1000 cm^−1^ while changing γ from 100, 200, 300, 400, and 500 cm^−1^. (**d**) Same key as for (**c**) but for the transmission spectra of 5.0 μm thick n-type GeC/Si(100) epifilm (see text).

At near-normal incidence (i.e., θ_i_ = 0°), we have displayed our simulated results (see Figure 7a,b) on a 5 μm thick GeC/Si (001) epifilm, signifying the impacts of γ and ω_P_ on $R\left(\omega \right)$ [$T\left(\omega \right)]$. The results of reflectance [transmission] spectra in Figure 7a,b are reported by using a constant value of γ = 150 cm^−1^ and varying ω_P_ from 300 to 1200 cm^−1^. Three significant changes are revealed (see Figure 7a,b) in the $R\left(\omega \right)$ [$T\left(\omega \right)$] spectra of GeC/Si (001) epifilm: (a) for lightly doped GeC film with ω_P_ (≡300 cm^−1^) or η ~1.6 E+18 cm^−3^, the first minimum [flat] in $R\left(\omega \right)$ [$T\left(\omega \right)$] appears at ~768 cm^−1^ just above$\;\omega_{\mathrm{LO}}$, (b) the reflectance [transmission] becomes much more pronounced [flat] as it shifts toward the higher frequency side with the increase in η or ω_P_, and (c) at low-frequency ω < $\omega_{\mathrm{TO}}\;$ the interference fringes nearly disappear while they have become smaller or even smeared out (1600 cm^−1^ > ω > $\omega_{\mathrm{LO}}$) with the increase in ω_P_ = 1200 cm^−1^ or η ~2.5 E+19 cm^−3^.

In Figure 7c,d, we have reported the results of $R\left(\omega \right)$ [$T\left(\omega \right)$] by using a constant value of ω_P_ = 1000 cm^−1^ and varying γ from 100 to 500 cm^−1^. Clearly, the frequency of $\omega_{\mathrm{LPP}\;}^{+}$ mode near ~1116 cm^−1^ for ω_P_ = 1000 cm^−1^ (or η ~1.7 E+19 cm^−3^) remains unaffected while the reflectance [transmission] spectra are changed as we increased the plasma damping constant γ from 100 to 500 cm^−1^.

#### 3.2.5. Berreman’s Effect

**Figure 8:**
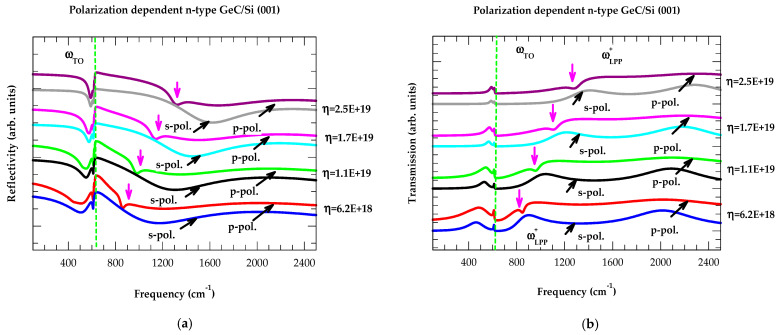
(**a**) Calculated infrared reflectivity spectra at oblique incidence (θ_i_ = 45°) for n-type GeC/Si (001) 1.0 μm thick film in the s- and p-polarization (different colors). The charge carrier concentration η increased from 6.2 E+18 cm^−3^ → 1.1 E+19 cm^−3^ → 1.7 E+19 cm^−3^ → 2.5 E+19 cm^−3^, respectively. The calculated shifts of $\omega_{\mathrm{LPP}\;}^{+}$ modes in the p-polarization spectra of GeC/Si are shown by the magenta-colored vertical arrows (see text). (**b**) Same key as for (**a**) but for the simulated transmission spectra of 1.0 μm thick epifilm with different charge carrier concentrations η.

In Figure 8a,b, we have displayed our calculated results of reflectivity and transmission spectra at oblique incidence (θ_i_ = 45°) in the s- and p-polarization for a ~1.0 μm thick n-type GeC/Si (001) epifilm having four different charge carrier concentrations η (i.e., 6.2 E+18 cm^−3^, 1.1 E+19 cm^−3^, 1.7 E+19 cm^−3^, and 2.5 E+19 cm^−3^). The results in p-polarized $R\left(\omega \right)\;$spectra have revealed a maximum at $\omega_{\mathrm{TO}}$ near ~625 cm^−1^ (see green-colored dotted line) with a dip at the higher frequency (shown by vertical magenta-colored arrows). With the increase in η, this dip clearly shifts from ~851 cm^−1^ → ~964 cm^−1^ → ~1116 cm^−1^ → ~1290 cm^−1^, respectively. In the s-polarized $R\left(\omega \right)\;$spectra, however, the simulations revealed only a maximum at $\omega_{\mathrm{TO}}$ near ~625 cm^−1^, irrespective of the values of η.

By increasing η, our revelation in the p-polarized $T\left(\omega \right)\;$spectra (see Figure 8b) with a minimum at $\omega_{\mathrm{TO}}$ ~625 cm^−1^ (green-colored dotted line) and a dip at higher frequency (vertical magenta-colored arrows) have also confirmed its shift from ~851 cm^−1^→ ~964 cm^−1^ → ~1116 cm^−1^ → ~1290 cm^−1^, respectively. Like $\left[R\left(\omega \right)\right],$ the study of $T\left(\omega \right)\;$ has perceived only one [maximum] minimum at $\omega_{\mathrm{TO}}$ near ~625 cm^−1^ in s-polarization, irrespective of the η values. Therefore, we assigned the high-frequency dips as $\omega_{\mathrm{LPP}}^{+}$ modes as they shift with the increase of η and emerge only in the p-polarization of the $R\left(\omega \right)\;$and $T\left(\omega \right)$ spectra in oblique geometry (Berreman effect) [121]. These outcomes have indicated that, like RSS, both $R\left(\omega \right)\;$and/or $T\left(\omega \right)$ at oblique incidence can provide alternative means of identifying $\omega_{\mathrm{LPP}}^{+}\;$modes. We strongly feel that these explorations in our findings are valuable for assessing the free charge carrier concentration η in thin n-doped GeC/Si (001) epifilms.

### 3.3. Modified Model for Infrared Spectra of Epifilms

In GeC/Si (001) epifilms, the assessment of film thickness, free charge carrier concentration, TL, and surface roughness can play critical roles in designing different micro/nanoelectronic device structures. Earlier on, different 3C-SiC/Si (001) epilayers [106,107,108,109,110] and extensive FTIR measurements have helped evaluate valuable information about the roughness at the film–surface and/or film–substrate interface, including the role of conducting TL. However, no such studies are available for the GeC/Si (001) epilayer structures.

Earlier, Pascual et al. [110] have shown that the surface roughness at 3C-SiC film can diminish the average reflectance of interference contrast with the increase in ω. They [110] have also indicated that film–substrate interface roughness may cause a decrease in the relative amplitudes of interference contrasts without affecting the average $\;\bar{R}\left(\omega \right)\;$value. In CVD-grown 3C-SiC/Si (001) epilayers, Holm et al. [106] measured $R\left(\omega \right)\;$spectra with different surface roughnesses, and it was suggested that rough surface can lead to distortion within the reststrahlen band region. By performing AFM measurements on ultrathin 3C-SiC/Si (001) epilayers with film thickness d (≡ 0.82 μm–1.29 μm), Dong et al. [109] have claimed to observe polycrystalline 3C-SiC domains, and within each domain the surface remains nearly flat.

Regarding the impact of film–substate interface roughness, the authors [109] have used cross-sectional SEM and observed a significant number of cavities produced by the interdiffusion of Si and C atoms during carbonization of Si substrate surfaces. While the existence of cavities at the interface can induce light scattering, the authors [109] did not observe their impact on the measured spectral profiles. One may note that the available theoretical analysis considering surface roughness in 3C-SiC/Si epilayers [106,107,108,109,110] is limited to being qualitative.

Our comprehensive measurements of the infrared reflectivity and transmission spectra on V-CVD-grown 3C-SiC/Si (001) samples with film thickness d (≡1.1 μm–20.0 μm) have revealed [60] atypical features exhibiting a decrease in fringe contrasts at higher ω > 2000 cm^−1^. In most samples, the $R\left(\omega \right)$ has seen the declining fringe inequalities with a drop in average ($\bar{R}$) value ranging between ~11 and 19%. Obviously, the ideal model (cf. Section 2.1) could not explain the unusual spectral traits. However, we have successfully appraised the observed atypical $R\left(\omega \right)$/$T\left(\omega \right)$ features in 3C-SiC/Si samples using a modified model.

#### 3.3.1. Modified Model for zb GeC/Si (001): Effects of δ and δ_2_

**Figure 9:**
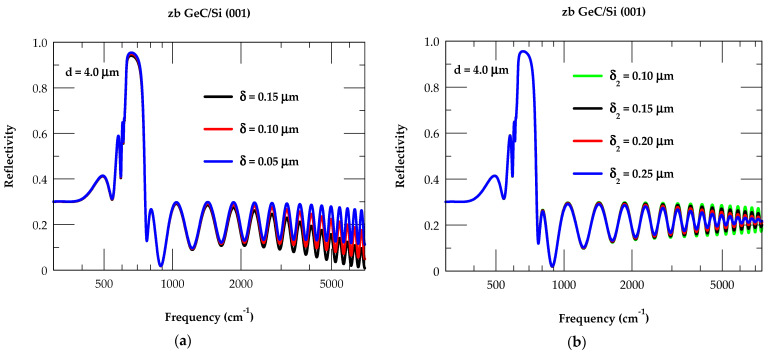
(**a**) Calculated reflectance at near-normal incidence for a 4 μm thick GeC/Si (100) epifilm (η~1.01 E+17 cm^−3^) with different air/film surface roughnesses δ (≡0.05 μm, 0.10 μm, and 0.15 μm). (**b**) Same key as for (**a**) but for different film/substrate interface roughnesses δ_2_ (≡0.10 μm, 0.15 μm, 0.20 μm, and 0.25 μm) (see text).

For GeC/Si (001) epilayers, we have reported (see Figure 9a,b) our calculated reflectivity results for a 4.0 μm thick film by employing a modified model (cf. Section 2.3). With δ = 0 (cf. Figure 3), the $R\left(\omega \right)$ spectrum of a 4.0 μm thick GeC epifilm has demonstrated the well-described interference fringe patterns. The effects of increasing δ [δ_2_] have revealed, however, high fringe contrasts below the reststrahlen band region (i.e., ω < 625 cm^−1^) and damping behavior at higher frequency (ω > 750 cm^−1^). The calculated $R\left(\omega \right)$ spectra displayed in Figure 9a with blue-, red-, and black-colored lines have considered air–film surface roughness δ ≡ 0.05 μm, 0.10 μm, and 0.15 μm [and in Figure 9b with green-, black-, red, and blue-colored lines of film–substrate interface roughness δ_2_ ≡ 0.10 μm, 0.15 μm, 0.20 μm, and 0.25 μm], respectively. Like in the ideal case, we have retained a lower value of η ~1.01 E+17 cm^−3^ in the modified model for simulating the $R\left(\omega \right)$ spectra with different surface and/or interface roughnesses. With δ = 0 (cf. Figure 3), the $R\left(\omega \right)$ spectrum of GeC epifilm demonstrated well-described interference fringe patterns. In the modified model, however, the effects of increasing δ have revealed a high fringe contrast below the reststrahlen band and the damping behavior away from the reststrahlen region (ω > 1600 cm^−1^), exhibiting the increased reduction with downward shifts of the interference fringes. Consistent with our GeC/Si (001) predictions, the observed reflectance $R\left(\omega \right)$ for the 3C-SiC/Si (001) epilayer with film thickness d (≡1.2 μm) and surface roughness δ (≡0.133 μm: rms value) has revealed [109] comparable trends.

For a 4.0 μm thick GeC/Si (001) epilayer, we have also reported our simulated reflectance $R\left(\omega \right)$ spectra (see Figure 9b) by carefully including GeC–Si interface roughness δ_2_ (≡0.10, 0.15, 0.20, and 0.25 μm). The fringe contrasts at frequencies above the reststrahlen band region in $R\left(\omega \right)$ are seen steadily decreasing with the increase in δ_2_. For δ_2_ (≡ 0.25 μm), the values become smaller, even diminishing at ω > 7500 cm^−1^ while retaining reflectance ~22%, as noticed in the semi-infinite GeC at higher ω. For GeC/Si (001) epilayers, our simulated trends in $R\left(\omega \right)$ agreed reasonably well with the experimental spectra of 3C-SiC/Si (001) epilayers [60,109]. In the epitaxially grown samples, we strongly feel this behavior is caused by optically rough (film–substrate) interfaces where the light scatters diffusely rather than reflecting perfectly.

#### 3.3.2. Modified Model for GeC/Si (001): Effects of δ, δ_2_, and TL Thickness

In strained epilayers (e.g., 3C-SiC/Si, GaN/sapphire, InN/Sapphire, etc.), many $R\left(\omega \right)$ and $T\left(\omega \right)$ measurements displayed damping behavior in the interference fringe contrast at higher photon energies. These damping traits are either associated with the formation of surface, interfacial roughness, and/or conducting ‘graphite-like’ TL between the film and substrate. There is a common belief that the relaxation of strain in these epilayer structures accompanies the formation of a high density of intrinsic defects, especially the dislocations. Again, in 3C-SiC/Si (001) epilayers, many TEM and AFM studies have confirmed the presence of a high density of defects near the interface as well as between the film and substrate [60,109]. As the observed damping behavior in interference fringes cannot be explained by exploiting an ideal model, attributing such damping to the formation of interfacial surface roughness $\delta_{2}\;$ and/or $\delta_{2}$+ $d_{2}\;$(TL) between film–substrate regions supports our assumptions made in the modified model.

**Figure 10:**
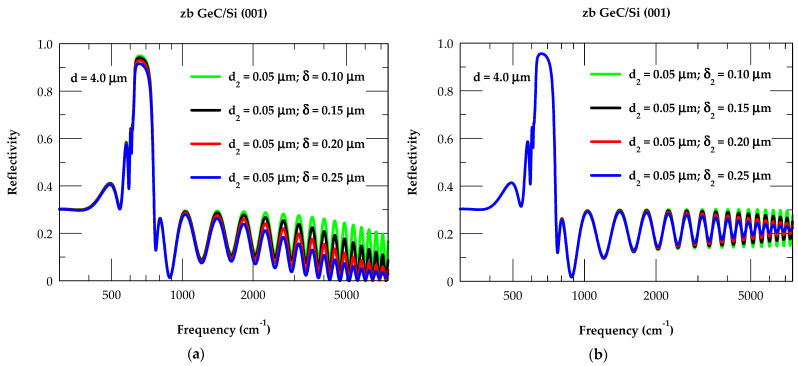
(**a**) Calculated reflectance spectra at near normal incidence (θ_i_ ≈ 0) for a 4 μm thick GeC/Si (100) epifilm (η~1.01 E+17 cm^−3^) for a fixed value of transition layer thickness${\;d}_{2}(\equiv \;$0.05 μm) and varying air/film surface roughness δ (≡ 0.10 μm, 0.15 μm, 0.20 μm, and 0.25 μm). (**b**) Same key as for (**a**) with a fixed value of transition layer thickness${\;d}_{2}(\equiv \;$0.05 μm) and varying film/substrate interface roughness δ_2_ (≡ 0.10 μm, 0.15 μm, 0.20 μm, and 0.25 μm) (see text).

By assuming the coexistence of TL $d_{2}(\equiv \;$0.05 μm) and surface [interface] roughness δ [$\delta_{2}](\equiv \;$0.10 μm, 0.15 μm, 0.20 μm, and 0.25 μm) in a modified model, we have simulated the reflectivity $R\left(\omega \right)\;$spectra of a 4.0 μm thick film of zb GeC/Si (001) epilayer. While the reflectivity results displayed in Figure 10a,b using green-, black-, red-, and blue-colored lines have exhibited behavior like those of Figure 9a,b, there are a few subtle differences. In agreement with the experimental observations in 3C-SiC/Si epilayers, the effects of (TL+δ) thickness on the $R\left(\omega \right)$ spectra for zb GeC/Si have exhibited (see Figure 10a) relatively sharp downward shifts (compared to Figure 9a in the interference fringes). The effects of fixed TL $d_{2}\;$and changing thickness of surface [interface] roughness δ [δ_2_] have exhibited a downward shift [reduction in interference fringe contrasts from 12% to 4% near ω ~5100 cm^−1^] in the reflectivity spectra.

## 4. Concluding Remarks

For novel GeC/Si (001) epilayers, we have reported the results of our comprehensive model-based simulations for IR reflectivity $R\left(\omega \right)\;$and transmission $T\left(\omega \right)\;$spectral profiles, at near-normal (θ_i_ ≈ 0) and oblique (θ_i_ ≠ 0) incidence. Different experimental [128] and theoretical methods (viz., Kirchhoff approach, finite element simulation, scalar diffraction, and Rayleigh–Rice theories) exist in the literature to study surface roughness in various materials [129,130]. While scanning probe microscopy and near-field optical microscopy are being used to obtain high-resolution optical images to study surface roughness and its impact on the optical properties in the NIR → UV region [129,130], their implementation is accompanied, however, with many difficulties and challenges (viz., probe fabrication, choosing optimal probe parameters, probing–surface distance, sensitivity of measurements to external parameters, high cost, etc.). Spectroscopic ellipsometry data in the NIR → UV region are required in the Rayleigh–Rice theory [129,130]. For qualitative investigation (especially in the absence of experimental data), it is preferred to consider a phenomenological approach for describing the surface properties. Here, we used a modified ‘Drude-Lorentz model’ for calculating $R\left(\omega \right)/T\left(\omega \right)\;$in the IR region for GeC/Si (001) materials of different epilayer thicknesses d and charge carrier concentrations η. The simulations have provided valuable information for assessing the role of surface roughness on film δ, film–TL interface δ_2_, and conducting TL thickness d_2_. Earlier analyses of IR reflectance/transmission spectra and the observations of SEM images in 3C-SiC/Si (001) have shown that the interfacial roughness and conducting layer are related to the carbonization process [96,109].

Our predicted results of $R\left(\omega \right)/T\left(\omega \right)\;$in GeC/Si (001) epilayers have revealed new features at low frequency (ω < 626 cm^−1^ ($\omega_{\mathrm{TO}}$)) while showing steady downward shifts and a reduction in fringe contrasts at higher frequencies (ω > 1600 cm^−1^). In undoped epilayers, the angle- and polarization-dependent simulations of reflectance [transmission] have established the Berreman effect [121], revealing (i) a sharp peak [dip] near $\omega_{\mathrm{TO}}\;$in both the s- and p-polarization and (ii) a second dip in p-polarization near the $\omega_{\mathrm{LO}}$ frequency. In n-doped epilayers, the calculated $R\left(\omega \right)\;$ [$T\left(\omega \right)]$ spectrum at oblique incidence has confirmed a peak [dip] near $\omega_{\mathrm{TO}}$ in p-polarization and an additional dip indicating an η dependent mode at a higher frequency. The high-frequency p-polarized mode shifts to higher values with the increase in η. As this mode, irrespective of η, did not appear in s-polarization, we therefore assigned it to $\omega_{\mathrm{LPP}}^{+}$, given its dependence on η and appearance only in the p-polarization. Based on this study, the Berreman effect in undoped and doped GeC/Si (001) epifilms has provided complementary information to Raman scattering spectroscopy, allowing the identification of $\omega_{\mathrm{LO}}$, $\omega_{\mathrm{TO}}$ phonons and LO-plasmon coupled $\omega_{\mathrm{LPP}}^{+}\;$modes, respectively. In the absence of experimental measurements, our systematic projections of the $(R\left(\omega \right)\;$and/or $T\left(\omega \right))$ spectral profiles have corroborated the results with the existing experimental studies on 3C-SiC/Si (001) epilayers [106,107,108,109,110]. We hope that this work on ultrathin films has the potential to offer a route in exploring the phonon characteristics for different novel GeC-based LDHs, as they are expected to impact many of their optical, electronic, and thermodynamical properties and help in designing different device structures for various technological applications. We strongly feel that the angle- and polarization-dependent predictions of $R\left(\omega \right)\;$and/or $T\left(\omega \right)$ profiles (Berreman effect [121]) in GeC/Si (001) might offer opportunities for spectroscopists to perform similar measurements to check our theoretical conjectures. Systematic calculations of the wave-vector-dependent spectral line shapes for LPP modes in n-doped XC/Si (001) epilayers are necessary for extracting accurate values of the charge carrier concentration η.

## Figures and Tables

**Table 1:** Optical constants of zb GeC and Si materials. The $\omega_{\mathrm{TO}},{\;\omega }_{\mathrm{LO}}\;$ modes and phonon damping Γ are in cm^−1^ for zb GeC and Si. These parameters are used in our calculations of IR reflectivity/transmission of zb GeC/Si (001).

Optical Constants for GeC and Si Material Parameters								
	$\varepsilon_{\infty }$	$\omega_{TO}$	$\omega_{LO}$	Γ	$m_{e}*$	$n_{s}$	$n_{f}$	
zb GeC (film)	7.70	626	749	4.5			2.7	Our
	7.29	626	748		0.20 $m_{e}$		2.7	Ref. [80] Ref. [81]
	7.20	630	755					Ref. [81]
	7.10	682	812					Ref. [86]
Si (substrate)	11.70	520	520			3.42		Refs. [77,78]

**Table 2:** Parameters and their values used for calculating the reflectivity/transmission spectra of n-doped GeC/Si (001) epilayers. The carrier concentration η (E+19 cm^−3^) dependence on $\omega_{P\;}$(cm^−1^), $\omega_{\mathrm{LPP}}^{-}$ (cm^−1^), and $\omega_{\mathrm{LPP}}^{+}$ (cm^−1^) are estimated from Figure 5 and Figure 6a,b.

Parameters	GeC									
η	0.019	0.07	0.16	0.28	0.43	0.62	0.84	1.1	1.7	2.5
$\omega_{P}$	100	200	300	400	500	600	700	800	1000	1200
$\omega_{\mathrm{LPP}}^{-}$	90	167	241	321	385	442	486	519	561	583
$\omega_{\mathrm{LPP}}^{+}$	750	756	768	787	813	851	901	964	1116	1290

## Data Availability

The data that support the findings of this study are available from the corresponding author upon reasonable request.
